# Investigating the efficacy of hyaluronic acid in minimizing black triangles: A comprehensive analysis

**DOI:** 10.12688/f1000research.158667.1

**Published:** 2024-12-04

**Authors:** Priyanka Paramita Sahu, Sangeeta Nayak, Ashita Uppoor

**Affiliations:** 1 Periodontology, Manipal College of Dental Sciences Mangalore, Manipal Academy of Higher Education, Manipal, Mangalore, Karnataka, 575001, India

**Keywords:** Dermal Fillers, Non-Invasive Treatment, Periodontal Aesthetics, Hyaluronic Acid, Black Triangles, Papilla Reconstruction, Interdental Papilla

## Abstract

In the anterior area of the mouth, the interdental papilla is important for dental hygiene and appearance. When it disappears, unpleasant “black triangles” form, which affects patients’ self-confidence in their smiles and makes oral hygiene more difficult. The loss of interdental papilla is caused by several variables such as tooth shape, periodontal disease, and aging. Although surgical treatments have been utilized to restore or retain missing papilla, their predictability remains unknown. In response, researchers have investigated non-invasive procedures, such as the use of fillers such as hyaluronic acid (HA).

Owing to its capacity to increase tissue volume and bind water, HA, a naturally occurring polysaccharide with special rheological qualities, has become a popular choice for use as a dermal filler. It shows promise when used to cure interdental papilla loss; the effects usually last for six–12 months. This review article explores the development and history of papilla rebuilding methods, emphasizing hyaluronic acid as a cutting-edge and successful method for regaining both periodontal health and aesthetics.

## Introduction

Restoring the lost periodontal support is one of the main goals of periodontal therapy. However, the interdental papilla exhibits a relatively limited ability to regenerate compared to other gingival segments. Effective black triangle management is crucial to uphold dental cleanliness while restoring patients who have lost confidence in their smiles.

The interdental papilla, the section of the free gingiva located between the teeth, constitutes only a small fraction of the visible surface area of oral tissues, both hard (teeth) and soft (gingiva and alveolar mucosa). Despite its modest physical size, the interdental papilla, which is almost always visible when smiling, has a disproportionately large impact on aesthetics. This is particularly true in the anterior region of the mouth. The significant worry around alterations in the proportions of the interdental papilla or its removal, which results in the formation of “black triangles,” emphasizes the papilla’s aesthetic significance. Several variables, including age, periodontal disease, crown form, root angulation, and position of interproximal contacts, cause the emergence of open gingival embrasures after the loss of the interdental papilla. ^1^

Drawing from morphological and histological studies, Cohen (1959) described the col as the buccal and lingual peaks of keratinized tissue with a non-keratinized or para-keratinized interproximal area. ^2^ The interproximal papilla and arrangement of the col were thoroughly studied by Matherson and Zander in their 1963 study. They discovered notable morphological variations between the anterior and posterior interdental papilla, indicating that the shape of the contact zone between neighboring teeth is reflected by the col rather than by the underlying interproximal bone. ^3^

Through the adoption of incision designs that either spare or preserve the existing papilla, attempts have been made to prevent the loss of the interdental papilla during intraoral surgical operations because of its aesthetic value. Although these surgical techniques are sophisticated and elegant, their predictability is unclear and is exceptionally dependent on the operators skills. Hence the use of non-surgical treatment modalities for the treatment of missing papilla has gained immense momentum recently.

Many fillers and biological preparations have been studied to restore the interdental papilla, either in conjunction with or independently of a concurrent access flap. Recently, hyaluronic acid gel compositions have been utilized as dermal fillers to treat interdental and interimplant papilla loss. Under physiological conditions, hyaluronic acid, a polysaccharide (glycosaminoglycan) present in bodily tissues such as the skin and cartilage, expands in gel form and binds to water, giving the appearance of smoother, fuller tissue outlines. N-acetylglucosamine and glucuronic acid disaccharide repeat to create a high-molecular-weight (≥10^5 Da) polymer backbone, which is made up of several thousand sugar molecules. ^4– 7^

Hyaluronic acid solutions have a viscosity that increases with concentration, and because of their special rheological characteristics, they are a perfect lubricant for use in biological applications. Hyaluronic acid preparations made through chemical changes, including cross-linking, break down more slowly because of their reduced water solubility. The clinical benefits of these fillers, which originate from animal or bacterial origins, usually last for six–12 months.

This narrative review explores the history and development of various treatment modalities for the reconstruction of interdental papilla, highlighting the role of hyaluronic acid in enhancing aesthetic outcomes and improving periodontal health.

## History and timeline

In 1961, Kohl and Zander studied the course and speed of interdental papilla healing in monkeys after the removal of interproximal tissue. They discovered that the papilla had grown back to its original shape and histological features by the eighth week following surgery. ^8^

Stahl (1963) showed that the degree of keratinization in the col area could be changed by applying interproximal stimulation. ^9^

Tarnow et al. examined the connection between the presence of the human interproximal papilla and the distance between the bone crest and contact point in a 1992 study. A total of 288 interproximal sites (99 anterior, 99 premolar, and 90 molar sites) across 30 patients who were selected at random were included in their study. They looked for any obvious gap apical to the contact point before probing, to determine whether the papilla was present or absent. Their findings demonstrated that when there was a distance of more than 7 mm between the bone crest and contact site, the papilla was typically absent. In contrast, the papilla is typically visible when the distance is 5 mm or less. ^10^

## Categorization of interdental papilla

**Figure 1.**
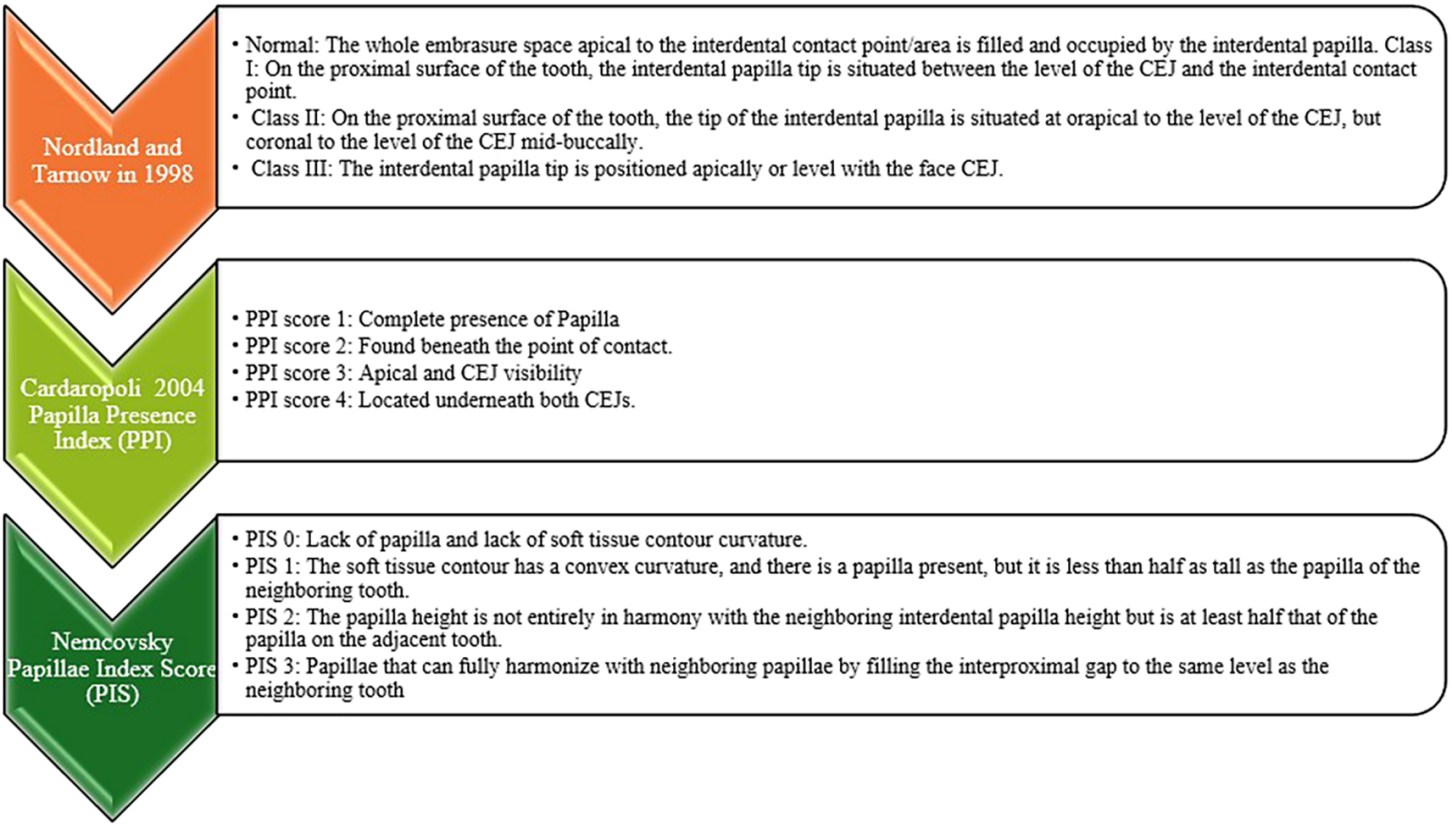
Categorization of interdental papilla.

The interdental papilla were classified using several indices ( Figure 1). According to Nordland and Tarnow (1998), the papilla fills the full embrasure space apical to the interdental contact point, which is considered to be normal. The papilla tip is between the CEJ and contact point in Class I deviations, the papilla tip is at or apical to the CEJ level but coronal to the CEJ mid-buccally in Class II deviations, and the papilla tip is at or apical to the face CEJ in Class III abnormalities. ^11^

The Papilla Presence Index (PPI), introduced by Cardaropoli (2004), assigns a score between 1 and 4 to the presence of papilla: PPI 1, total presence; PPI 2, beneath the contact point; PPI 3, apical and CEJ visibility; and PPI 4, beneath both CEJs. ^12^

The Nemcovsky’s papilla Index Score (PIS) ranges from 0 to 3. A papilla height of less than half that of the neighboring tooth is indicated by PIS 1; a convex curvature with a papilla less than half that of the neighboring tooth is indicated by PIS 2; a papilla height that is at least half that of the neighboring tooth but not fully harmonious is indicated by PIS 3; and a fully harmonizing papilla that fills the interproximal gap to the same level as the neighboring tooth is indicated by PIS 4. ^13^

## Understanding the interdental papilla function

Tiny gingival tissue, the interdental papilla, is located between the teeth, significantly impacting oral health and appearance. A well-sculpted papilla can enhance the appearance of a smile by properly outlining each tooth, maintaining fullness, and ensuring balance. A balanced and appealing smile depends on the presence of this microscopic structure. ^14^

Beyond appearance, the papilla plays a crucial role in maintaining healthy gingiva by preventing bacterial growth and trapping food particles, thereby lowering the risk of periodontal illnesses and gingival inflammation. During mastication, it shields the periodontal tissue and bone underneath from potential injury or mechanical stress. Gingival tissue is well supplied with blood owing to its robust vascular network. This vasculature promotes healing and maintains tissue health through its role in tissue regeneration and repair. ^15^

The papilla aids in the immune response of the periodontium by combating infection and microbial invasion. The interdental papilla significantly contributes to healthy gingiva, supports blood vessels, ^16^ and bolsters the immune system.

## Determinants influencing the existence of papilla

**Figure 2.**
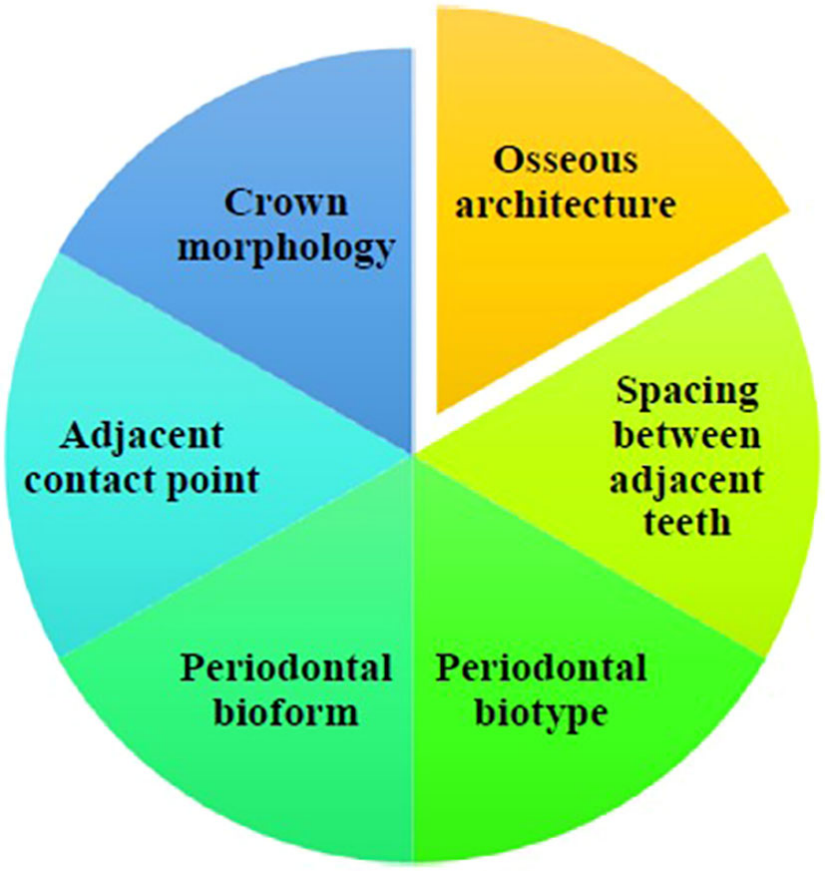
Determinants influencing the existence of papilla.

Several factors affect the size and shape of the interdental papilla; however, the underlying bone architecture is one of the most important. The osseous crest usually guarantees a favorable architecture by imitating the geometry of the cement-enamel junction. The coronal nature of the interproximal bone has a major impact on the development of the papilla ( Figure 2). According to Tarnow et al. (1992), the papilla is present 98% of the time when there is a 5 mm gap or less between the alveolar crest and the contact point, and it becomes much less frequent beyond that. ^17^

The distance between neighboring teeth is also crucial; Tal (1984) ^18^ stated that to maintain the interdental papilla, there must be a minimum of 3 mm between roots. Thin periodontal biotypes affect papilla healing and resilience. The thick biotype contributes to improved long-term papilla maintenance, because it is more resilient and resistant to recession.

Periodontal bioform or gingival scallop shape influences surgical results as well as appearance. While high scallops can produce “black triangles” due to mismatched gingival and bone forms, flat scallops precisely correspond with the bone structure, limit recession risks, and improve cosmetic outcomes. ^19, 20^

The interdental papilla is also affected by the crown morphology. The papilla is better supported by square-shaped crowns because they have larger contact areas and are closer to the bone crests. However, there is an increased risk of papilla recession in cases of triangular crowns because of their thinner crestal bones. ^21^

Finally, the position of the nearby contact point was important. Tarnow’s ‘5 mm rule’ states that the papilla fills the gingival embrasures if there is a 5 mm gap or less between the contact point and bone crest. ^22^ Compared to triangular teeth with narrow incisally positioned contact points, teeth with a square form and wider contact points have a lower likelihood of developing black triangles. ^23, 24^

## Treatment modalities for black triangle

**Figure 3.**
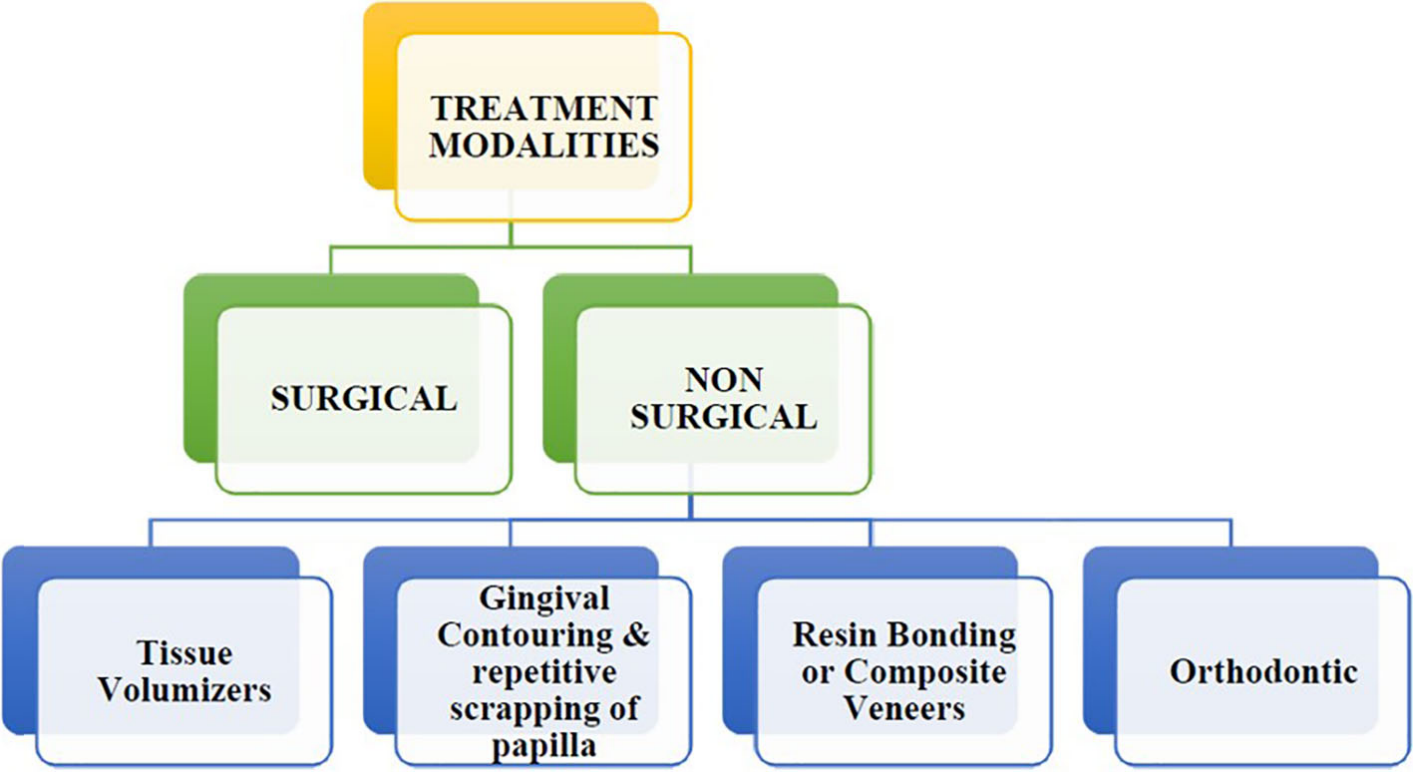
Treatment modalities for black triangle.

SURGICAL TECHNIQUES FOR RECONSTRUCTION OF PAPILLA ( Figure 3)

a) Gingivectomy: This procedure reshapes the gum line for better appearance and function by removing extra gum tissue, particularly in cases of drug-induced or idiopathic hyperplasia.
b) Papilla Reconstruction: To repair interdental papilla, palate tissues are used in surgical procedures, such as pedicle grafting (Beagle, 1992). Using a periodontal dressing to promote healing, this technique involves severing a flap from the palatal side and suturing it to form a new papilla. ^25^
c) Semilunar Coronally Repositioned Papilla: This method was first described by Han and Takie (1996). It involves filling the interdental area with connective tissue grafts to sustain and improve the papillary structure. ^26, 27^
d) Envelope Technique: To restore the volume and shape of the interdental papilla, Azzi et al. (1998) described wrapping a connective tissue graft with an envelope-style flap that is carefully placed. ^26^

Extended follow-up periods and larger sample sizes are necessary to improve our understanding of the predictability and long-term usefulness of the various surgical methods. By addressing the drawbacks of conventional techniques, non-surgical treatment strategies aim to provide more accurate and possibly more successful papilla repair treatments in restorative dentistry.

Non-surgical options: Black triangles are more common with orthodontic treatments, such as braces and aligners, because they straighten teeth and close gaps. The contact areas are widened by enamel loss, and new papilla are formed by mild pressure from orthodontics. Veneers and resin bonding fill the gaps, making the teeth look better. Gums are reshaped by gingival contouring and papilla regeneration is encouraged by periodic curettage. Hyaluronic acid injections fill spaces and promote tissue regeneration in the short term. Maintaining dental hygiene through consistent use of interdental brushes and flossing lowers the visibility of black triangles.

## Revolutionising oral aesthetics: HA bridging the divide of black triangle

HYALURONIC ACID (HA) is a naturally occurring marvel bestowed upon by nature. It has gained immense popularity in plastic surgery because of its remarkable characteristics. HA, an essential constituent of connective tissues with remarkable qualities, has gained significant attention in plastic surgery owing to its role in promoting skin hydration, elasticity, and joint lubrication. Plastic surgery is utilized for renewing the skin appearance, volumizing, and shaping contours. Plastic surgeons employ HA’s biocompatible and non-immunogenic qualities for non-invasive or minimally invasive facial enhancements, such as face augmentation and dermal fillers. This option, known for its transitory effects and adaptability, is frequently chosen for cosmetic procedures, resulting in precise modifications and natural outcomes aligned with patients’ aesthetic desires.

In recent years, the reconstruction of the interdental papilla has emerged as a popular topic in dental practice and research. Among various treatments, HA therapy has proven to be a promising choice for papilla regeneration. HA offers advantages for interdental papillary repair. It is suitable for intraoral use owing to its biocompatibility, non-immunogenicity, and quick absorption by oral tissues. HA possesses wound healing and anti-inflammatory properties, which are essential for promoting tissue regeneration in the periodontal region. HA-based therapies effectively improve the shape and size of interdental papilla by encouraging tissue regeneration upon application or injection into the papillary region. ^28^

In 2010, **Becker et al**. conducted a pilot study to address cosmetic concerns with papillary deficits next to teeth or dental implants. These deficiencies can have a major effect on the overall appearance of a smile. An exploratory technique was used to determine how well an HA gel sold commercially could reduce or eliminate these inadequacies. There were seven females and four males (average age, 55.8 years) in the cohort who participated in the study. Among them, 14 sites were treated with papillary defects in the aesthetic zone. Using a 23-gauge needle positioned apically at the coronal tip of the afflicted papilla, precise injection of less than 0.2 mL of HA gel was administered as part of the therapy regimen. This study determined the percentage change in the black triangle space associated with papillary deficiency. A significant enhancement was reported in all treated sites, ranging from 57% to 97%, which was sustained during the follow-up period. ^29^

**Mansouri et al. (2013)** concluded a study emphasized the difficulty of reconstructing the interdental papilla, especially in the aesthetic zone. Eleven patients with 21 interdental papillary deficits were included in the study. A 0.2cc or less of HA gel was administered per location following local anesthesia. At 3-week and 3-month follow-up, the same procedure was repeated. After more than six months, the interdental papilla was successfully reconstructed because of the gel’s ability to promote angiogenesis, migration, and proliferation. It decreased scar tissue formation and increased collagen production, basal keratinocyte growth, and re-epithelization. 10 % of the participants showed a 50% improvement in interdental papilla repair at the second follow-up. 43% of the participants showed even better results after 6 months of follow-up. Differences were considered statistically significant at p<0.05. Hence, it was concluded that HA gel has acceptable long-term outcomes. ^30^

In **2016, Awartani et al.** assessed the feasibility of restoring anterior teeth aesthetics with lost interdental papilla using injected HA gel. Ten adults were included in the trial; all had at least one anterior location showing a loss of class I or II interdental papilla. To achieve the best results, the patients were injected with HA gel directly into the base of their papilla twice at baseline 21-day and 42-day intervals. A 23-gauge needle was used to injecting 0.2 ml the cross-linked HA gel. The region was gently massaged for a minute following the injection. The positive response of the patients and the notable progress in papilla restoration indicated that HA gel injection could be a promising treatment option for aesthetic issues related to interdental papilla loss. ^31^

**Lee et al. in 2016** studied the radiographic anatomical factors influencing papillary reconstruction and assessed the clinical efficacy of HA gel injections to enhance insufficient interdental papilla. Periapical radiography and photography were standardized for each patient using specific equipment to ensure reliable assessment. The patients were observed for six months post-treatment. The computer software simplified the clinical measurements of the black triangle area, height, and width. Contact point-to-bone crest (CP-BC) and interproximal root distances were assessed using periapical radiography. Nearly full papillary reconstruction was achieved when the CP-BC distance reached 6 mm. This means that the effectiveness of HA gel injection for interdental papilla repair depends on the role of CP-BC. ^32^

**Singh et al. (2019)** studied the economic feasibility of using 1%, 2%, and 5% HA injectable gels for treating IDP. For three weeks, the papillary tip received weekly injections of HA 2 mm apical to it. At the one-, three-, and six-month marks, IDP augmentation was assessed using the UNC-15 probe, a modified stent, and ImageJ software for analysis. The 5% HA group showed a statistically significant improvement compared to the group treated with other concentrations. This suggests that the 5% HA gel was not only economically feasible but also yielded superior clinical outcomes. ^33^

**Alhabashneh’s (2020)** evaluated the effectiveness of HA in treating IDP in the aesthetic zone. 0.2 ml HA injections were administered to each IDP site at two intervals 21 days apart. These HA formulations, traditionally derived from bacterial or animal sources, provide a standard therapeutic effect lasting between six and 12 months. A clear, biodegradable, animal-free HA gel, HYADENT BG, was introduced for the restoration of the interdental papilla. HA was injected using a three-step procedure following the manufacturer’s instructions. The needle was angled at 45 °with its bevel against the bone. 21 days later, the injections were administered in the same manner. Patients were assessed three and six months after the initial HA injection. The study suggested that commercially available HA gel yielded the most significant improvement in restoring interdental papilla during the initial six months of therapy, peaking at the three-month mark after injection. ^34^

**Ni et al. in 2021**, evaluated the effectiveness of HA in conjunction with physiological saline solution in restoring deficient gingival papilla in vitro and in vivo. in vitro investigations assessed the migration and proliferation of gingival fibroblasts after HA and saline treatment. It was observed that HA administration at 1%, 3%, and 6% concentration for 3-4 days led to a higher gingival fibroblast proliferation rate in the CCK -8 assay. In the cell migration assay, gingival fibroblasts exhibited approximately 13% greater migration than the control group. This suggests that HA stimulates fibroblasts, suggesting its potential for tissue regeneration and leads to the enhancement of gingival papillary defects (35).

## Conclusion

The “black triangle” is formed by gum recession or bone loss that can cause both functional and aesthetic problems for the individuals. The presence of these gaps in a person’s dentition can impair the health of their gums and affect their confidence in smiling. Patients might avoid social situations because of their self-consciousness of their smiles. Proper periodontal therapy is essential for enhancing oral hygiene and promoting patients’ overall health by effectively addressing the black triangles. Recognizing the limited capacity of the interdental papilla for regeneration is essential to enhance treatment outcomes and increase patient satisfaction.

HA injections have gained popularity as a non-surgical, minimally invasive solution for addressing aesthetic concerns, allowing patients to enhance their smiles and boost their self-confidence. HA injection enhances the strength of weak interdental papilla, reduces the appearance of black triangles, and improves smile aesthetics. Clinical research has shown that HA effectively enhances gingival symmetry and improves papillary aesthetics. HA injections between teeth create a more natural and aesthetically beautiful smile by seamlessly connecting neighboring teeth. The benefits of HÀ injection surpass those of traditional black-triangle treatments. Unlike surgical procedures, which require prolonged healing times and have potential risks, an HA injection can be administered in a dentist’s office with minimal invasiveness. Patients usually experience minimal, temporary swelling and bruising at the injection point with minimal discomfort. The durability of the HA injection results is an important consideration for patients undergoing black triangle therapy. Depending on the formulation and concentration, HA fillers yield results that last for a minimum of six–12 months. Numerous studies have shown that HA injections lead to high levels of patient satisfaction along with improvements in overall well-being and self-confidence. The effectiveness of HA in dental applications depends on its local delivery to the target area within the oral cavity. Challenges related to adherence and extended release arise in the creation of oral delivery systems, such as gels, films, or scaffolds. It is essential to study the immunogenic reactions to HA to ensure safer and more effective use at higher dosages or over longer periods.

The use of hyaluronic acid (HA) in dentistry presents numerous challenges. Owing to the burgeoning interest, more rigorous trials and long-term investigations are required to provide robust clinical evidence for HA’s efficacy and safety of HA across various dental procedures. The extensive variety of hyaluronic acid formulations and molecular weights hampers consistent standardization, hindering the definitive evaluation of its effectiveness. In dental settings, new methods are required to ensure the long-term durability and biodegradability of HA without compromising its therapeutic effectiveness. Precise techniques are needed for the effective local delivery of HA to ensure targeted distribution within the oral cavity. Although infrequent, immunogenic reactions necessitate further studies to address the potential harmful consequences. Obtaining regulatory approval for HA-based dental products requires multi-stakeholder cooperation. Effective communication regarding the benefits, risks, and outcomes of HA-based therapies is crucial for fostering acceptance and understanding among patients. Addressing these challenges is essential for maximizing HA’s impact of HA in advancing dental treatments and improving patient care. As John Muir correctly observed, ***"Between every two pines is a doorway to a new world."***

As the quote implies, HA injections provide a reliable and efficient solution for addressing gaps, such as those between teeth, depicted as black triangles. HA injections improve patients’ smiles by enhancing periodontal health and papillary aesthetics and ensuring the effectiveness of treatment plans. With ongoing research and the resolution of challenges, HA will soon replace other methods such as the standard black triangle treatment, showcasing its transformative effect within our smiles.

## Ethics and consent

Ethical approval and consent were not required.

## Data Availability

No data are associated with this article.
