# Supplementary material for: Investigating the efficacy of hyaluronic acid in minimizing black triangles: A comprehensive analysis
Source: F1000Res. 2025 Jun 19;13:1481. Originally published 2024 Dec 4. [Version 2] doi: 10.12688/f1000research.158667.2 (PMC12290441; doi:10.12688/f1000research.158667.2)
Supplement: Peer review history

# Reviewer report: Reviewer response for version 2

**DOI:** 10.5256/f1000research.183520.r393275

**Published:** 2025-07-24

**Authors:** Álvaro Ferrando Cascales

**Affiliations:** 1 Universidad Católica San Antonio de Murcia (UCAM), Murcia, Spain

The article now provides a clearer contextual framework.

Is the review written in accessible language?

Yes

Are all factual statements correct and adequately supported by citations?

Yes

Are the conclusions drawn appropriate in the context of the current research literature?

Partly

Is the topic of the review discussed comprehensively in the context of the current literature?

Partly

Reviewer Expertise:

Esthethic and restorative dentistry

I confirm that I have read this submission and believe that I have an appropriate level of expertise to confirm that it is of an acceptable scientific standard.

# Reviewer report: Reviewer response for version 1

**DOI:** 10.5256/f1000research.174297.r386633

**Published:** 2025-06-03

**Authors:** Álvaro Ferrando Cascales

**Affiliations:** 1 Universidad Católica San Antonio de Murcia (UCAM), Murcia, Spain

**1. Is the topic of the review discussed comprehensively in the context of the current literature?**

**Answer:** ***Partly***

 The review provides a useful summary of the etiology of interdental papilla loss and the use of hyaluronic acid (HA) as a non-invasive treatment. However, the discussion of alternative regenerative or surgical techniques is limited. The article mentions classic techniques like pedicle grafts or semilunar coronally repositioned papilla but omits updated regenerative protocols involving enamel matrix derivatives (e.g., Emdogain®), bone graft substitutes, or the use of growth factors, which are well-supported in the current literature for papilla reconstruction. Furthermore, restorative approaches such as the injection molding technique with composite resin (Bioclear method) are not mentioned, even though they offer predictable and minimally invasive management of black triangles. These omissions limit the comprehensiveness of the review.

**4. Are the conclusions drawn appropriate in the context of the current research literature?**

**Answer:** ***Partly***

 While the manuscript provides a general overview of hyaluronic acid (HA) as a non-surgical approach for interdental papilla reconstruction, it fails to incorporate or critically compare this approach with well-established regenerative techniques in periodontal therapy. Notably, key references addressing the use of enamel matrix derivatives (e.g., Emdogain®), connective tissue grafting, or guided tissue regeneration are missing. For instance, the consensus report by **Jepsen S, Caton JG, Albandar JM, Bissada NF, Bouchard P, Cortellini P, et al.**

*Periodontal manifestations of systemic diseases and developmental and acquired conditions: Consensus report of workgroup 3 of the 2017 World Workshop on the Classification of Periodontal and Peri‐Implant Diseases and Conditions.* **J Clin Periodontol.** 2018;45(Suppl 20):S219–S229. offers substantial evidence regarding surgical papilla reconstruction protocols and should have been discussed. Additionally, While this review emphasizes the use of hyaluronic acid (HA) for the reconstruction of interdental papillae, it does not acknowledge the comparative regenerative potential of enamel matrix derivative (EMD), such as Emdogain®, which is supported by recent clinical trials. For instance, a 2025 randomized controlled clinical trial ( *Clinical Oral Investigations*, doi: 10.1007/s00784-025-06278-5) directly compared crosslinked HA with EMD and found that EMD achieved superior clinical outcomes in periodontal tissue regeneration. Although the study did not specifically target papilla reconstruction, its findings strongly support the inclusion of EMD in discussions on papillary tissue regeneration. The absence of such comparative data in the present review limits its comprehensiveness and clinical relevance.

 In addition to hyaluronic acid and surgical approaches, restorative techniques using injectable composite resins have shown significant promise in black triangle management. For example, Ferrando et al. (2023) demonstrated that the injection molding technique (Bioclear Method) can achieve stable and esthetic interdental papilla reconstruction by mimicking natural emergence profiles without the need for surgery. This technique not only addresses the black triangle but also restores anatomical and functional contour predictably. Including such restorative alternatives would strengthen the review’s comparison of minimally invasive approaches and provide clinicians with broader evidence-based options.The absence of these comparative approaches limits the clinical relevance and comprehensiveness of the review. Furthermore, although the authors acknowledge the short duration of HA's effects (typically 6–12 months), they do not provide any forward-looking recommendations for long-term randomized trials or comparative efficacy studies. For a narrative review aiming to inform clinical decision-making, such omissions should be addressed to ensure a balanced and up-to-date representation of current therapeutic options.

Is the review written in accessible language?

Yes

Are all factual statements correct and adequately supported by citations?

Yes

Are the conclusions drawn appropriate in the context of the current research literature?

Partly

Is the topic of the review discussed comprehensively in the context of the current literature?

Partly

Reviewer Expertise:

Esthethic and restorative dentistry

I confirm that I have read this submission and believe that I have an appropriate level of expertise to confirm that it is of an acceptable scientific standard, however I have significant reservations, as outlined above.

# Reviewer report: Reviewer response for version 1

**DOI:** 10.5256/f1000research.174297.r368058

**Published:** 2025-02-28

**Authors:** Biju Thomas

**Affiliations:** 1 AB Shetty Memorial Institute of Dental Sciences, NITTE (Deemed to be University), AB Shetty Memorial Institute of Dental Sciences, Mangaluru, India

This article Investigating the efficacy of hyaluronic acid in minimizing black triangles: A comprehensive analysis, explores the development and history of papilla rebuilding methods, with special  emphasis on hyaluronic acid as a cutting-edge and successful method for achieving both periodontal health and aesthetics. The background , history and timelines are clearly highlighted. The importance of having a interdental papilla in esthetics and function and the factors influencing it are explained. The various surgical techniques for the management currently available are also highlighted.

 The advantages of the use of hyaluronic acid and its potential future role are highlighted.

 Last sentence HA will soon replace other methods can be modified as there is a possibility of HA replacing other methods.......

Is the review written in accessible language?

Yes

Are all factual statements correct and adequately supported by citations?

Yes

Are the conclusions drawn appropriate in the context of the current research literature?

Yes

Is the topic of the review discussed comprehensively in the context of the current literature?

Yes

Reviewer Expertise:

clinical periodontology, stem cell applications in periodontology .

I confirm that I have read this submission and believe that I have an appropriate level of expertise to confirm that it is of an acceptable scientific standard.
